# Stress-Strain Behavior and Strength Development of High-Amount Phosphogypsum-Based Sustainable Cementitious Materials

**DOI:** 10.3390/ma17194927

**Published:** 2024-10-09

**Authors:** Ying Shi, Yue Li, Hongwei Wang, Yixuan Ma, Xinyue Lu

**Affiliations:** School of Resources and Safety Engineering, Central South University, Changsha 410083, China

**Keywords:** solid waste, cementitious material, stress–strain behavior, compressive strength

## Abstract

Phosphogypsum is a common industrial solid waste that faces the challenges of high stockpiling and low utilization rates. This study focuses on the mechanical properties and internal characteristics of cementitious materials with a high phosphogypsum content. Specifically, we examined the effects of varying amounts of ground granulated blast furnace slag (5–28%), fly ash (5–20%), and hydrated lime (0.5–2%) on the stress–strain curve, unconfined uniaxial compressive strength, and elastic modulus (E_50_) of these materials. The test results indicate that increasing the ground granulated blast furnace slag content can significantly enhance the mechanical properties of phosphogypsum-based cementitious materials. Additionally, increasing the fly ash content can have a similar beneficial effect with an appropriate amount of hydrated lime. Furthermore, microscopic analysis of the cementitious materials using a scanning electron microscope revealed that the high sulfate content in phosphogypsum leads to the formation of calcium aluminate as the main product. Concurrently, a continuous reaction of the raw materials contributes to the strength development of the cementitious materials over time. The results could provide a novel method for improving the reusing phosphogypsum amount in civil engineering materials.

## 1. Introduction

Ordinary Portland Cement (OPC), as a common cementitious material, is in high demand in the road and construction industries. However, the cost of OPC production and the accompanying environmental pollution have been unresolved issues for OPC as a cementitious material. The cement industry generally requires 1.2 tonnes of hydrated limestone and 0.3 tonnes of clay to produce one tonne of cement clinker [1]. In the process of producing one tonne of OPC, 700 kg of carbon dioxide is emitted into the atmosphere [2,3,4,5,6], as well as other toxic and harmful gasses [7,8,9,10]. Therefore, many scholars are committed to researching and developing low-cost green cementitious materials that can replace OPC [11,12,13]. Industrial production generates a large amount of solid waste [14,15,16,17]. These solid wastes, at present, are mainly disposed of at landfills with low comprehensive utilization [18,19,20,21,22]. Therefore, preparing low-cost green cementitious materials using various types of solid wastes is a pathway with great potential for development [23,24,25,26,27,28].

Phosphogypsum (PG), as a typical industrial solid waste, is generally produced as a by-product from the production of phosphoric acid. It is primarily composed of calcium sulfate dihydrate, along with impurities such as soluble phosphorus, fluorine, and heavy metals [29,30]. It was reported that approximately five tonnes of PG are generated in the production of one tonne of phosphoric acid. The global production of PG is about 300 million tonnes per year [31,32]. Its mass utilization would be 10–15% under ideal conditions [33]. The use of PG to prepare cementitious materials has been studied [34,35,36]. Min et al. [37] used PG, FA, lime, and cement to prepare cementitious materials. They found that relying on the interaction between the raw materials, they could obtain a cementitious material with excellent comprehensive performance. Xu et al. [38] mixed phosphonyls with iron tailings to prepare cementitious materials. They concluded that phosphonyls could effectively promote the generation of calcium alumina in the cementitious system, fill the large pores in the system, and significantly improve the compressive strength of cementitious materials. Liu et al. [39] aimed to address the issues of long setting time and low early strength in gelling agents. They explored a novel approach by adding polymerized aluminum compounds to regulate the nucleation and growth of calcite. This research developed a low-carbon gelling agent based on waste gypsum and metallurgical slag. Zhang et al. [40] prepared a low-carbon calcium sulphoaluminate cement by replacing natural gypsum with PG. They discussed the mechanical properties and hydration mechanism of this new cement.

Previous studies show that although PG can provide a large amount of sulfate, it lacks the silicon and aluminum elements needed for hydration reactions [41]. Therefore, the synergistic reaction with substances containing more silicon and aluminum elements mixed with PG [42] can effectively enhance the mechanical properties of PG-based cementitious materials. However, at present, most studies use low-volume (<45%) PG to partially replace OPC as composite cementitious materials [43,44,45,46]. There is still a knowledge gap in using high-volume (50–90%) PG to fabricate cementitious material without OPC. Therefore, this study planned to prepare low-cost cementitious materials using high-volume PG without OPC. Additionally, other solid wastes containing some amount of silicon and aluminum elements, i.e., ground granulated blast furnace slag (GGBS), and fly ash (FA), are required. GGBS effectively enhances the strength growth of the cementitious material in the early stage, while FA ensures the strength increase in the later stage of the cementitious material. At the same time, PG can raise sufficient amounts of sulfate to facilitate the volcanic ash reaction process during this period.

The properties of the relevant cementitious materials in different aspects are represented by a number of other parameters [47,48,49], while the mechanical properties are generally characterized by parameters such as peak strength, stress–strain curves, E_50_, etc. [50,51]. Referring to previous scholars’ research on cementitious materials [52,53,54]. The following exploration of test results was carried out in this paper: (1) analyzing the effect of changes in the components of the cementitious materials on the mechanical properties of paste; (2) studying the relationship between compressive strength and E_50_ of paste; (3) determining the microstructure of paste based on the scanning electron microscope micrographs.

## 2. Experimental Program

### 2.1. Raw Materials

**Figure 1:**
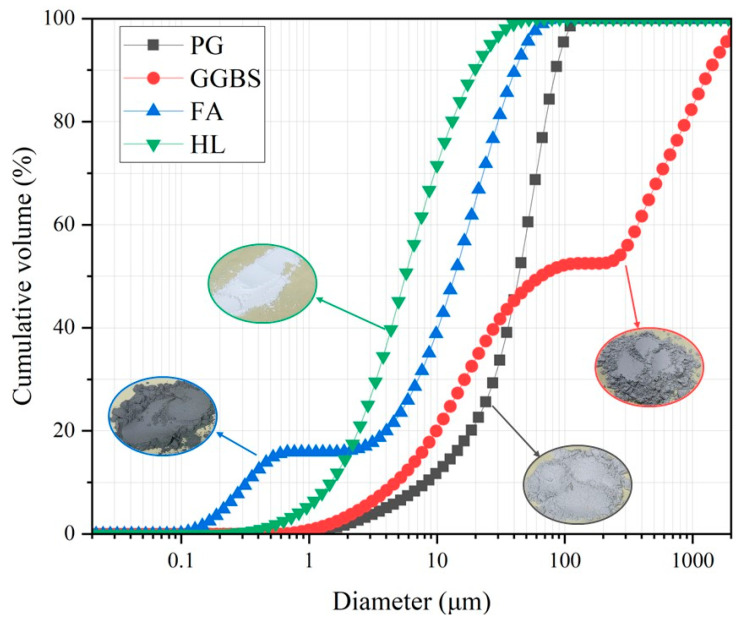
Particle size analysis of raw materials.

The PG used in this study was sourced from Xifeng, Guizhou, China. Prior to use, it underwent water washing to remove attached pollutants. Based on pre-experimental research, the mass ratio of PG to water was 1:3, with stirring conducted for a total of half an hour. The GGBS, with S95-grade activity, was procured from the Baifeng Mineral Products Processing Plant, Shijiazhuang, China. F-grade FA was obtained from Longjiang FA Development and Utilisation Co. (Heilongjiang, China). Hydrated lime (HL) from Shanghai McLean Biochemical Technology Co. (Shanghai, China) was added to the reaction system to enhance the volcanic ash reaction. The particle size analysis of PG, GGBS, and FA was conducted using a Mastersizer 3000 laser diffraction particle size analyzer, with the results illustrated in Figure 1. The d50 values for PG, GGBS, FA, and HL were 50.5 μm, 73.7 μm, 15.2 μm, and 4.9 μm, respectively.

**Figure 2:**
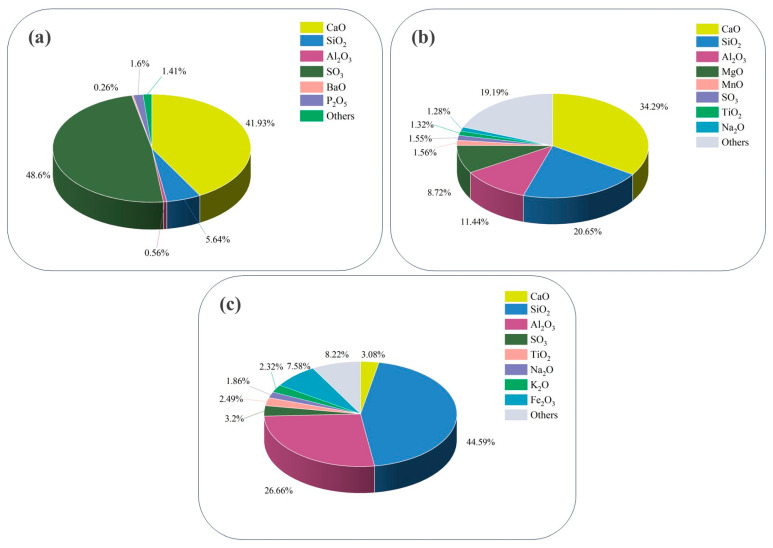
XRF analysis of raw materials: (**a**) PG; (**b**) GGBS; (**c**) FA.

In this study, an X-ray fluorescence spectrometer (XRF) was used to analyze the main chemical constituents of solid waste. The results are shown in Figure 2. The main components of PG were 41.9% CaO and 48.6% SO_3._ GGBS was mainly composed of 34.3% CaO, 20.6% SiO_2_, and 11.4% Al_2_O_3_. In contrast, FA was composed of 44.6% SiO_2_ and 26.7% Al_2_O_3_.

### 2.2. Test Method

To examine the effects of three raw materials on the mechanical properties of PG-based paste specimens, the experimental design involved fixing the content of two raw materials while varying the content of the third. The raw material mixing for the cementitious materials was designed based on findings from previous laboratory studies [55]. Using the two groups G-1 and G-2 from Table 1 as examples, this study explores the impact of varying GGBS content on the properties of the cementitious materials, while maintaining constant levels of FA and HL. Given that PG, at a dosage greater than 50%, can serve as both a calcium source and a provider of sulfate ions, its individual variation is not considered in this research [55]. Table 1, Table 2 and Table 3 provide specific content information for each group. Based on previous laboratory findings, the water-to-binder ratio of paste was fixed at 0.5.

**Figure 3:**
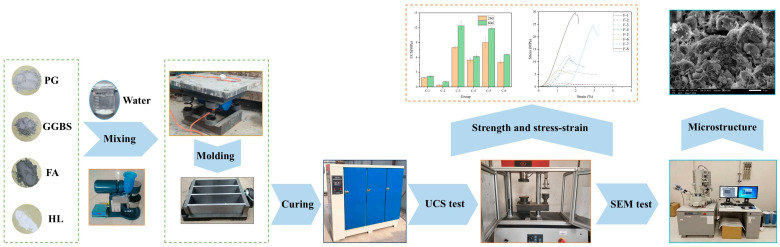
Specimen preparation process and test.

Figure 3 illustrates the specimen preparation process and testing apparatus. Firstly, dry PG, GGBS, FA, and HL were mixed for two minutes using a planetary mixer to obtain a homogeneous mixture. A corresponding mass of water was then added and mixed for another two minutes. Subsequently, the mixture was poured into 40 mm × 40 mm × 160 mm molds, and the poured molds were placed on a shaking table to eliminate air bubbles. Afterward, cling film was attached to the surface of the molds and maintained for three days before demolding. The specimens were further wrapped with cling film and cured in a constant temperature and humidity box (SHBY-60B, Cangzhou Yixuan Testing Instrument, Cangzhou, China) at 20 °C and 95% humidity.

The specimens’ unconfined compressive strength (UCS) at 28 days and 60 days was determined using a WHY-200/10 (Cangzhou Yixuan Testing Instrument) microcomputer-controlled compression tester. The corresponding data were analyzed to obtain the stress–strain curves. The UCS tests in this study refer to the Chinese standard GB/T17671 [56]. The uniaxial compressive strength result was the average of the test results of three specimens.

After the compressive strength test, specimens with a volume of 5 mm^3^, featuring natural fractures and flat surfaces, were selected. These specimens were dried in a constant-temperature oven at 60 °C until they reached a constant weight. Prior to testing, the samples were coated with gold foil to enhance conductivity. Scanning electron microscope images of these samples were obtained using a Phenom ProX (Thermo Fisher Scientific, Waltham, MA, USA) scanning electron microscope with an accelerating voltage of 10 KV.

## 3. Result and Discussion

### 3.1. Stress and Strain

The corresponding stress–strain curve data for each group were exported and fitted to the curve when using the WHY-200/10 microcomputer-controlled compression tester to test the uniaxial compressive strength. The horizontal axis represents the axial strain of the cementitious material, while the vertical axis represents the axial stress of the cementitious material. Based on the change in stress rate, the stress–strain curve of the specimen is divided into four stages: compaction stage, elastic deformation stage, plastic deformation stage, and post-peak damage stage. In the compaction stage, the specimen closes the pores due to external pressure, resulting in a slow increase in stress with strain [57]. The elastic deformation stage is entered after the pores are closed, characterized by an approximately linear change in stress with strain. Subsequently, as the specimen continues to deform and the internal pores and cracks expand, it transitions into the plastic deformation stage under high pressure [58,59]. At this time, the stress growth rate of the specimen will continue to decline, reducing to zero when the peak stress is reached. Continuing to apply pressure to the specimen at this point will cause it to enter the post-peak damage stage. In this stage, the bearing capacity of the specimen will be reduced, and obvious cracks will appear on the surface [60].

**Figure 4:**
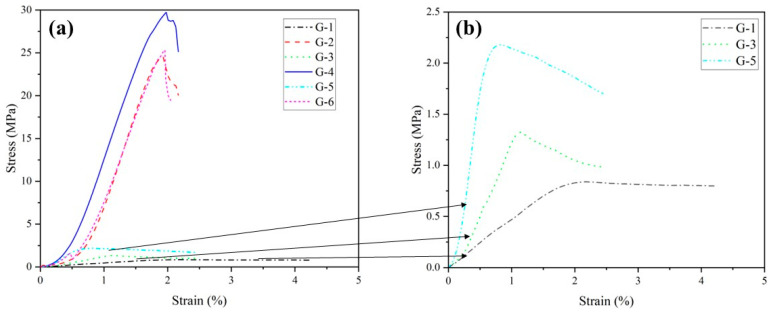
(**a**) Stress–strain relationship for different GGBS content levels; (**b**) is an enlargement of the typical curves in (**a**).

Figure 4 illustrates the stress–strain curves for each group in Table 1 at 60 days. From the figure, it is evident that in the group with higher FA content, a higher GGBS content shortens the compaction stage of the specimen. This is because GGBS contains a certain amount of cement clinker and a significant amount of reactive silica-alumina, both of which fully participate in the hydration reaction [61]. This suggests that a significant amount of hydration products is generated inside the specimen to fill numerous pores. When the content of FA and HL is fixed, increasing the GGBS content in cementitious materials markedly enhances the slopes of the stress–strain curves in both the elastic and plastic deformation stages. This indicates a notable positive effect of increasing GGBS content on improving the deformation resistance of cementitious materials. Additionally, specimens in the group with a higher GGBS content ratio exhibited damage at higher strain values and demonstrated more ductile behavior.

**Figure 5:**
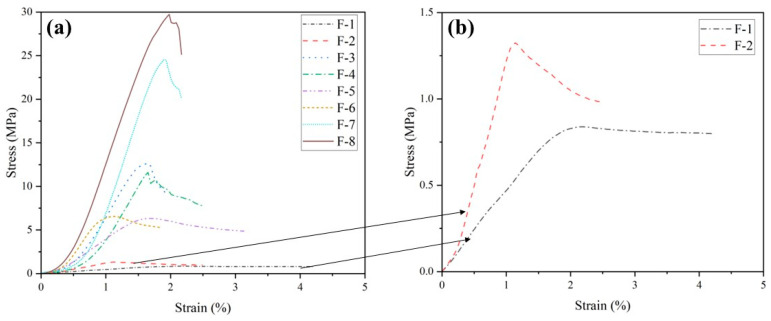
(**a**) Stress–strain relationship for different FA content levels; (**b**) is an enlargement of the typical curves in (**a**).

Figure 5 illustrates the stress–strain curves for each group in Table 2 at 60 days. At higher GGBS and HL dosages, increasing the FA dosage significantly shortens the compaction stage of the cementitious materials. This indicates that sufficient amounts of GGBS and HL greatly enhance the participation of FA in the reaction, leading to the generation of more hydration products that fill internal pores. In contrast, with low GGBS and HL dosages, increasing the FA dosage prolongs the compaction stage of the specimens, suggesting that FA requires adequate alkalinity to dissolve the vitreous surface, thereby enabling the internal reactive silica-alumina to participate in the reaction [62]. After entering the ascending branch of the elastic deformation stage and the plastic deformation stage, increasing the FA content was able to increase the slope, indicating that FA participated in the reaction system in all groups and improved the deformation resistance of the specimens. However, the elevation of FA content decreases the failure strain values of the specimens, indicating a gradual transition from ductile to brittle specimens.

**Figure 6:**
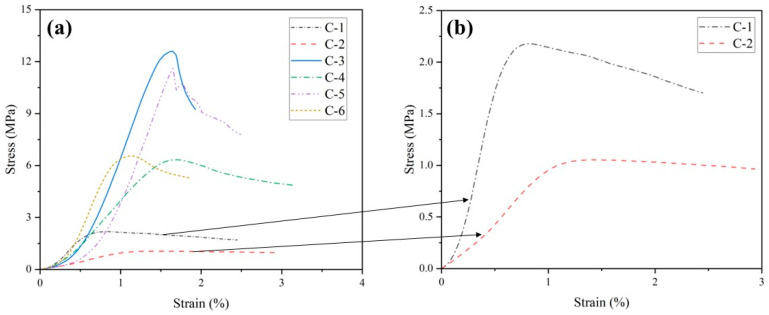
(**a**) Stress–strain relationship for different HL content levels; (**b**) is an enlargement of the typical curves in (**a**).

Figure 6 illustrates the stress–strain curves at 60 days for the groups in Table 3. From the figure, it can be seen that the compaction stages of the group with high HL content are all lower than those of the group with low HL content, indicating that HL can effectively neutralize the residual acid of PG and promote the participation of GGBS and FA in the reaction [63] so that there are enough hydration products inside the specimen to fill the pores. If the GGBS and FA content is low, the slopes of the group with high HL content will be lower than that of the group with low HL content in both the elastic deformation stage and plastic deformation stage, indicating that too much HL will be harmful to the deformation resistance of the specimen. However, if the content of GGBS and FA is increased, the HL will be fully utilized, increasing the specimens’ deformation resistance. The effect of HL content on the failure strain of the specimen is related to the content of other raw materials. Based on the high content of GGBS and FA, increasing the content of HL will make the specimen reach the failure strain value earlier and show higher brittleness. However, if the HL content is increased based on low GGBS and FA content, the specimen reaches the failure strain value later and shows higher ductility.

### 3.2. Compressive Strength

**Figure 7:**
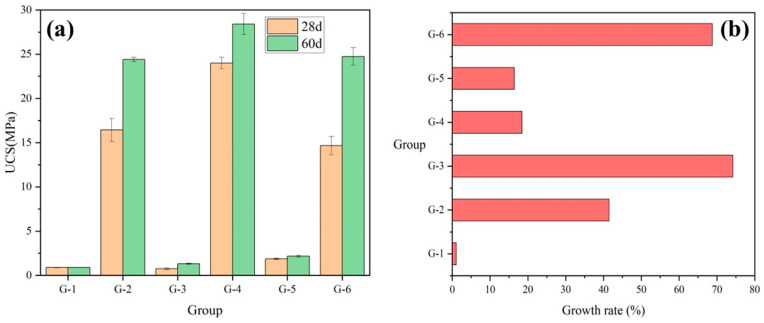
Effect of GGBS content on (**a**) UCS and (**b**) growth rate.

Figure 7a demonstrates the UCS at 28 and 60 days for each group of Table 1. Figure 7b demonstrates the growth rate of each group’s strength at 60 days relative to 28 days for different GGBS content, calculated as shown in Equation (1). It can be observed that the strength of specimens with high GGBS content is significantly higher than that of specimens with low GGBS content, regardless of the variation in FA and HL content. This is attributed to the fact that GGBS contains a certain amount of cement clinker and a large amount of active silica-alumina, effectively enhancing the strength of cementitious materials [61]. The presence of high GGBS content can effectively ensure the strength growth value of the specimens from 28 days of maintenance to 60 days. The strength growth rate of the high GGBS content group was higher than that of the low GGBS content group, except for G-4. The strength growth rate in the G-3 group was 74%, significantly higher than the 18% observed in the G-4 group. This difference is attributed to the much higher initial strength value of the G-3 group at 28 days. The G-4 group formulations outperform the others in this study.

(1) $$Growth\;rate\left(\%\right)=\frac{{UCS}_{60d}-{UCS}_{28d}}{{UCS}_{28d}}$$

**Figure 8:**
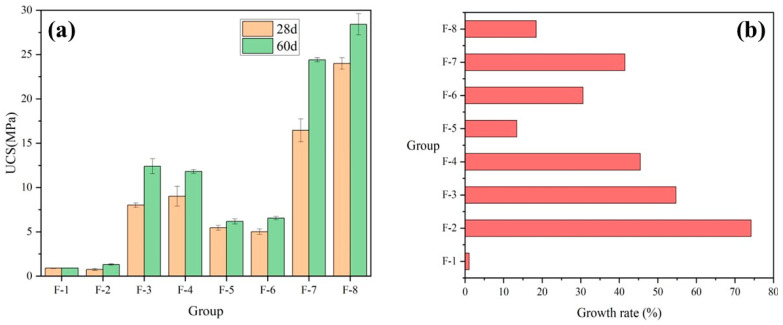
Effect of FA content on (**a**) UCS and (**b**) growth rate.

Figure 8a illustrates the UCS of each group of Table 2 at 28 and 60 days. Figure 8b illustrates the growth rate of each group’s strength at 60 days relative to 28 days for different FA contents. As shown in the figure, while keeping the low HL content constant, the strength of the high FA content group (F-4) is lower than that of the low FA content group (F-5). Conversely, the strength of the high FA content group is higher than that of the low FA content group for all other ratio groups, indicating that to promote FA’s participation in the reaction, it is necessary to ensure a certain level of alkalinity [64]. Thus, due to high alkalinity, the strength growth rate of 30% in the high FA content group (F-6) was significantly higher than the 13% strength growth rate in the low FA content group (F-5).

**Figure 9:**
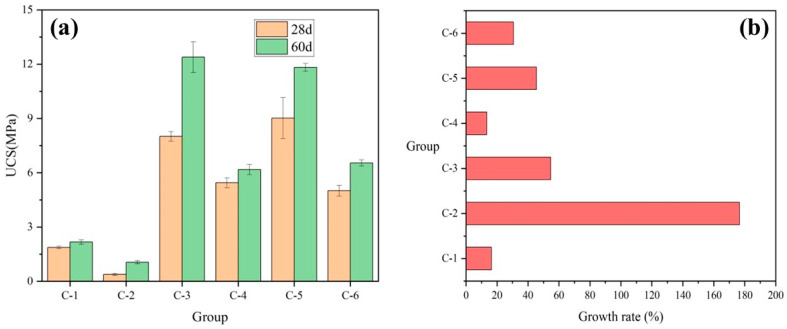
Effect of HL content on (**a**) UCS and (**b**) growth rate.

Figure 9a illustrates the UCS of each group of Table 3 at 28 and 60 days. Figure 9b illustrates the growth rate of each group at 60 days strength relative to 28 days strength for different HL contents. It can be seen from the figure that the high HL admixture has a negative effect on the hydration reaction inside the specimen [65]. The strengths of the specimens in the high HL content group are much lower than those in the low HL content group. While keeping the low content of GGBS and FA unchanged, the strength growth rate of the high HL content group (C-2) was 176%, which was 16% higher than the strength growth rate of the low HL content group (C-1). Conversely, other groups exhibit the opposite trend: the C-4 strength growth rate of 13% is lower than the C-3 strength growth rate of 54%, and the C-6 strength growth rate of 30% is lower than the C-5 strength growth rate of 45%. This suggests that the low lime content group still requires a certain amount of GGBS to undergo hydrolysis reaction [66], generating HL crystals to maintain the alkalinity of the reaction system.

### 3.3. Prediction Model

As shown in Table 4 below, the different dosages of raw materials used in this study were grouped into three level values. A reasonable prediction model was constructed using UCS at a curing time of 60 days as an example.

The corresponding simplified mathematical model is shown in Equation (2). Table 5 displays the statistical indicators of the constructed model, including essential metrics such as the F-value, *p*-value, and R^2^.

(2) $$UCS\left(60d\right)=9.89+10.88\;A+0.63\;B-2.63\;C+1.26\;AB-1.82\;AC+0.23\;BC\;+2.75\;A^{2}+0.91\;B^{2}-1.57\;C^{2}$$

The F-value for the model in Table 5 is 28.17, indicating that the model is significant. The model has a probability of 0.09%, and the observed large F-value is not attributed to noise. In this study, the *p*-value for the model is less than 0.05, affirming the significance of the model. Meanwhile, the R^2^ value of the model is 0.9807, indicating that the model accurately represents the actual situation [67,68].

**Figure 10:**
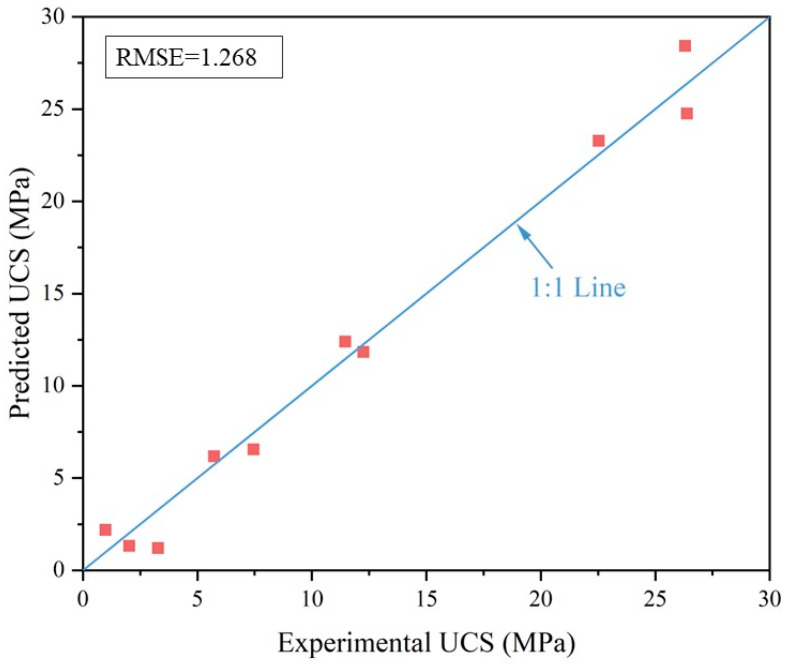
Comparison of experimental and predicted UCS.

The comparison of experimental and predicted UCS is shown in Figure 10. Using the model fitted to the test data, the UCS values for a curing time of 60 days can be predicted. The predicted values are generally comparable to the experimental values, with a root mean square error (RMSE) of only 1.268 MPa. Consequently, the reliability of the fitted model is considered high.

Based on the cementitious materials prepared in this study, two experimental groups with different ratios were created. A compressive strength test was conducted after 60 days of curing. The results were used to verify the accuracy of the previously constructed model, as shown in Table 6. The findings demonstrate that the model possesses a notable degree of generalizability.

### 3.4. Elastic Modulus

The E_50_ is a constant of proportionality of material over the range of elastic deformation and is a parameter that clearly reflects the mechanical properties of the prepared cementitious material [69,70]. In this study, the constant of proportionality of the prepared cementitious material at 50% peak stress is taken and calculated as shown below:

(3) $$E_{50}=\frac{\sigma_{50}}{\varepsilon_{50}}$$

where $E_{50}$ is the constant of proportionality of the cementitious material at 50% peak stress, MPa. The $\sigma_{50}$ is the 50% peak stress of the cementitious material, MPa. The $\varepsilon_{50}$ is the the strain value at the 50% peak stress of the cementitious material.

**Figure 11:**
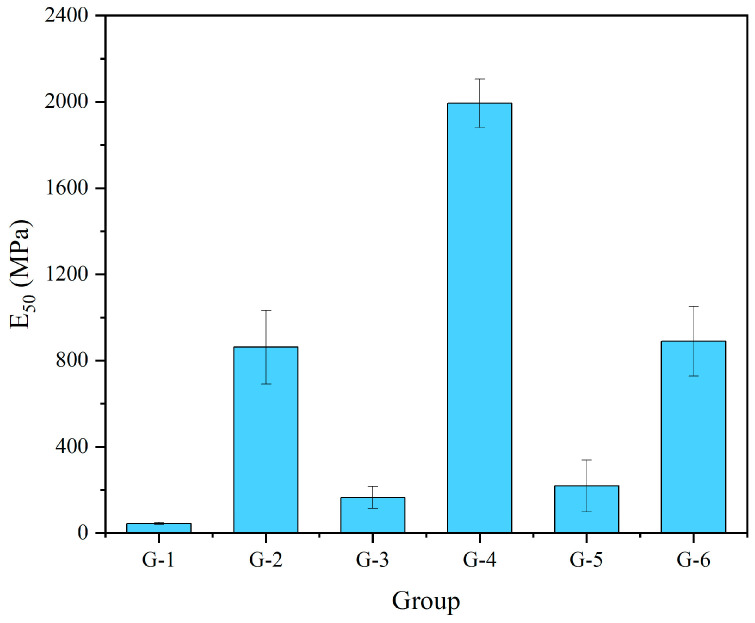
E_50_ for different GGBS content groups.

The following Figure 11 shows the E_50_ for different GGBS content groups. Elevated GGBS content significantly increases the E_50_ of the specimens, with the highest group, G-4, reaching an E_50_ of 2000 MPa, nearly 12 times higher compared to the corresponding low GGBS content group G-3.

**Figure 12:**
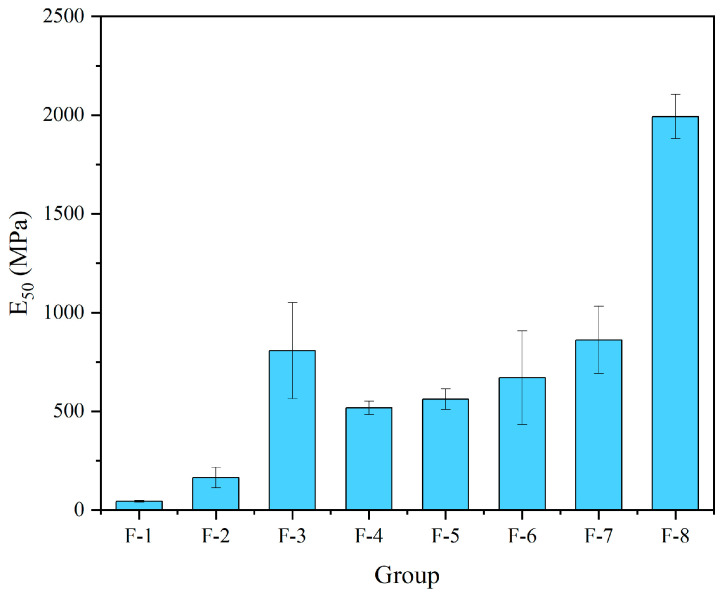
E_50_ for different FA content groups.

The following Figure 12 shows the E_50_ for different FA content groups. Based on low HL content, increasing the FA content decreases the E_50_ of the specimens (F-3 vs. F-4). However, increasing the FA content in the remaining groups significantly increases the E_50_, which is consistent with the change in the strength of the specimens. The highest of these groups, F-8, improved nearly three times compared to F-7.

**Figure 13:**
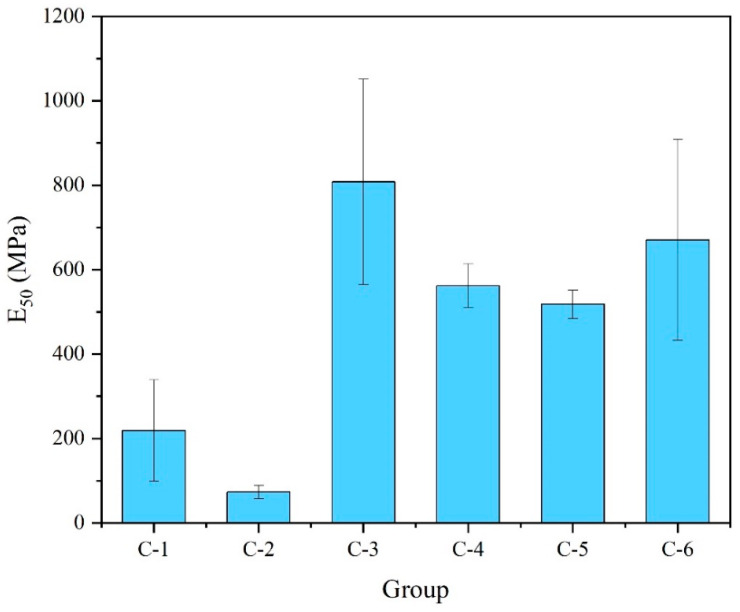
E_50_ for different HL content groups.

The following Figure 13 shows the E_50_ for different HL content groups. Based on high FA content, increasing the HL content enhances the E_50_ to some extent (C-5 vs. C-6), which is related to the need for a certain alkalinity of the FA. Whereas, increasing the HL content for the other groups significantly reduces the E_50_.

**Figure 14:**
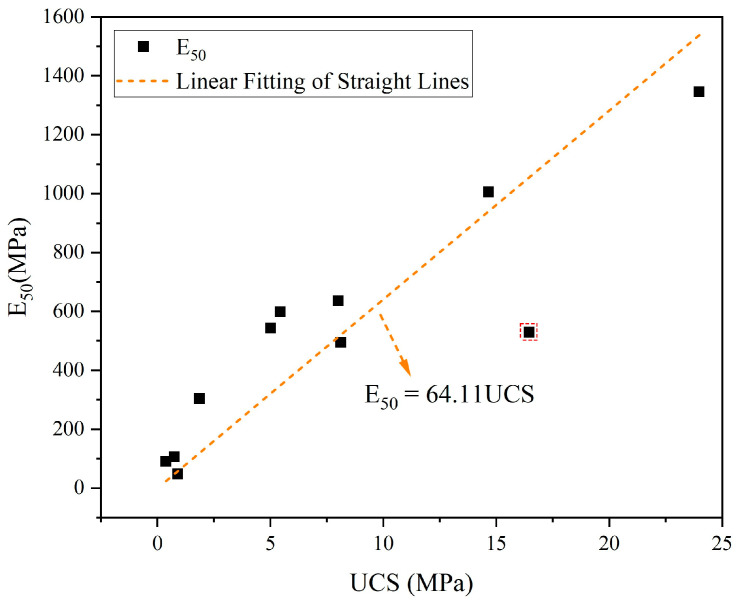
Relationship between E_50_ and UCS.

Based on the UCS and E_50_ discussed above, the corresponding data for each group were curve-fitted to build the model equations, as shown in Figure 14 (data points identified with dashed boxes in the figure are discrete points). The E_50_ of each group has a gradually increasing trend with UCS, and the higher the UCS, the larger the E_50_. The corresponding linear correlation equation is E_50_ = 64.11UCS, and the correlation coefficient of the equation is R^2^ = 0.95, which is highly reliable.

### 3.5. SEM

**Figure 15:**
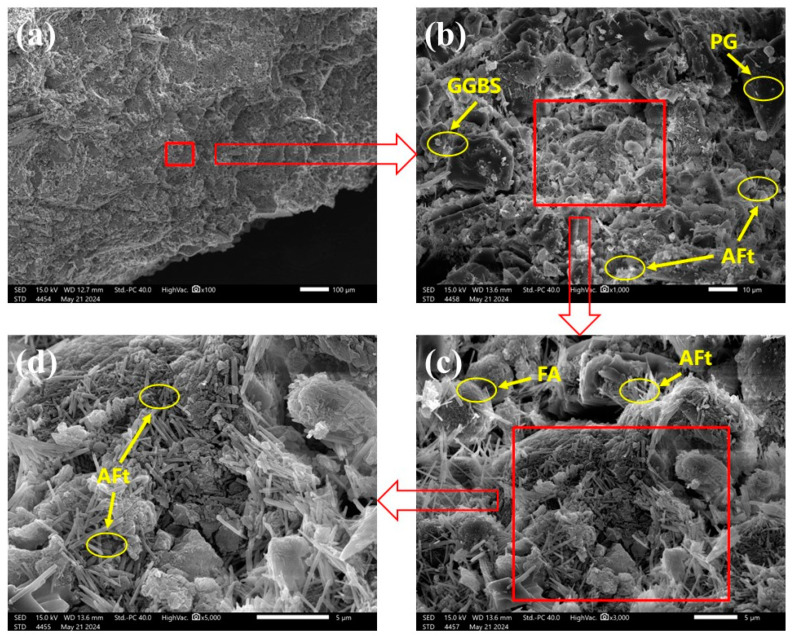
SEM images of typical specimens. (**a**) specimen (×100); (**b**) is a local enlargement of (**a**) (×1000); (**c**) is a local enlargement of (**b**) (×3000); (**d**) is a local enlargement of (**c**) (×5000).

In this study, the typical microstructure of this system was analyzed using the optimized proportioning group based on the strength results as a representative specimen. The results are shown in Figure 15. From Figure 15d, it can be seen that the main product of the reaction of the cementitious materials is ettringite, which is associated with the high sulfate content provided by PG [25]. As shown in Figure 15b,c, a certain amount of PG, GGBS, and FA is still present within the collodion material, indicating that the hydration reaction has not been fully completed in a short period. In the later stages of the hydration reaction, the glassy layer on the surface of the FA gradually dissolves. This dissolution releases reactive silica-aluminum compounds [71,72]. These reactive substances react with the abundant sulfates in the system, producing adequate amounts of ettringite to fill the pores within the cementitious material. Consequently, the cementitious material is expected to exhibit significant strength growth in the later period (60 days).

## 4. Conclusions

This study analyzes the mechanical properties and microstructure of a PG-based cementitious material developed using various solid wastes as raw materials to improve the reusing of PG in civil engineering materials. Different raw materials’ effects on the cementitious material’s properties are clarified, and relevant fitting models are constructed. The main conclusions are as follows:

(1) A high GGBS content (28%) can compensate for the pores inside the cementitious material and improve deformation resistance. Similarly, a high FA content (20%) can achieve the same function with sufficient alkalinity. However, excessive alkalinity negatively affects the material’s performance.
(2) High GGBS content significantly improves the UCS of cementitious materials. Moreover, increasing FA content under appropriate HL conditions also enhances UCS.
(3) A prediction model was constructed based on the UCS of cementitious materials. The statistical indices of the constructed model indicate a certain degree of reliability, and the model’s predicted data are generally consistent with the test data.
(4) The pattern of change in E_50_ and UCS of cementitious materials tends to be the same, so the relationship between the two is modeled as a linear equation, represented as E_50_ = 64.11UCS.
(5) SEM results indicate that ettringite primarily fills the pores within the studied cementitious material. As the curing age increases, the unreacted materials in the system gradually participate in the hydration reaction, continuously producing ettringite to further fill the pores of the cementitious material.

## Figures and Tables

**Table 1:** The ratio of experimental groups with different content of GGBS.

No.	GGBS (%)	FA (%)	HL (%)	PG (%)	Total (%)
G-1	5	5	1.25	88.75	100
G-2	28	5	1.25	65.75	100
G-3	5	20	1.25	73.75	100
G-4	28	20	1.25	50.75	100
G-5	5	12.5	0.5	82.00	100
G-6	28	12.5	0.5	59.00	100

**Table 2:** Ratio of experimental groups with different content of FA.

No.	GGBS (%)	FA (%)	HL (%)	PG (%)	Total (%)
F-1	5	5	1.25	88.75	100
F-2	5	20	1.25	73.75	100
F-3	16.5	5	0.5	78	100
F-4	16.5	20	0.5	63	100
F-5	16.5	5	2	76.5	100
F-6	16.5	20	2	61.5	100
F-7	28	5	1.25	65.75	100
F-8	28	20	1.25	50.75	100

**Table 3:** Ratio of experimental groups with different content of HL.

No.	GGBS (%)	FA (%)	HL (%)	PG (%)	Total (%)
C-1	5	12.5	0.5	82	100
C-2	5	12.5	2	80.5	100
C-3	16.5	5	0.5	78	100
C-4	16.5	5	2	76.5	100
C-5	16.5	20	0.5	63	100
C-6	16.5	20	2	61.5	100

**Table 4:** Range of variation in prediction model factors.

Variables	Coded	Levels		
		−1	0	1
GGBS/wt.%	A	5	16.5	28
FA/wt.%	B	5	12.5	20
HL/wt.%	C	0.5	1.25	2

**Table 5:** The model fitting parameters.

Curing Time	Mean	F-Value	*p*-Value	R^2^
60 d	11.01	28.17	0.0009	0.9807

**Table 6:** Model validation.

GGBS (%)	FA (%)	HL (%)	PG (%)	Predicted UCS (MPa)	Experimental UCS (MPa)
28.00	12.50	2.00	57.50	17.50	16.33
16.50	12.50	1.25	69.75	9.89	10.13

## Data Availability

The authors confirm that the data supporting the findings of this study are available within the article.
